# ISMB 2008 Toronto

**DOI:** 10.1371/journal.pcbi.1000094

**Published:** 2008-06-27

**Authors:** Michal Linial, Jill P. Mesirov, B. J. Morrison McKay, Burkhard Rost

**Affiliations:** 1 International Society for Computational Biology (ISCB), University of California San Diego, La Jolla, California, United States of America; 2 Sudarsky Center, The Hebrew University of Jerusalem, Jerusalem, Israel; 3 Broad Institute of MIT and Harvard, Cambridge, Massachusetts, United States of America; 4 Department of Biochemistry and Molecular Biophysics, Columbia University, New York, New York, United States of America

The International Society for Computational Biology (ISCB) presents the Sixteenth International Conference on Intelligent Systems for Molecular Biology (ISMB 2008), to be held in Toronto, Canada, July 19–23, 2008. Now in the final phases of scheduling selected presentations, demonstrations, and posters, the organizers are preparing what will likely be recognized as the premier conference on computational biology in 2008. ISMB 2008 (http://www.iscb.org/ismb2008/) will follow the road paved by the ISMB/ECCB 2007 (http://www.iscb.org/ismbeccb2007/) in Vienna in the attempt to specifically encourage increased participation from previously under-represented disciplines of computational biology. This conference will feature the best of the computer and life sciences through a variety of core sessions running in multiple parallel tracks, along with single-tracked *Keynote Presentations*, posters on display throughout the duration of the conference, and an extensive commercial exposition. The first day (July 18) of the meeting is reserved for two-day *Special Interest Group* (SIG) and *Satellite* meetings, the second day (July 19) runs SIGs for the first time in parallel with *Tutorials* and the *Student Council Symposium*, and for the first time two SIGs are running in parallel with the main ISMB meeting (July 20–23).

## Introduction

A considerable fraction of all the major scholars in computational biology frequently participate in ISMB. Consistent with this leading role in representation, ISMB has become a major outlet for increasing the visibility of this extremely dynamic new discipline, and for maintaining and elevating its scientific standards. It has become the vehicle for the education of scholars at all stages of their careers, for the integration of students, and for the support of young leaders in the field. ISMB has also become a forum for reviewing the state of the art in the many fields of this growing discipline, for introducing new directions, and for announcing technological breakthroughs. ISMB and ISCB are contributing to the advance of biology, and to helping to build bridges and understanding between dedicated and passionate groups of scholars from an unusual variety of backgrounds.

### 

#### ISMB 1993–2008

The ISMB conference series began in 1993, the result of the vision of David Searls (GlaxoSmithKline), Jude Shavlik (University of Wisconsin Madison), and Larry Hunter (University of Colorado). A few years later, ISMB had established itself as a primary event for the computational biology community and triggered the founding of ISCB, the International Society for Computational Biology (http://www.iscb.org/). ISCB has been organizing the ISMB conference series since 1998. While ISCB evolved into the only society representing computational biology globally, its flagship conference has become the largest annual worldwide forum focused on computational biology. In January 2007, the ISCB came to an agreement with the European Conference on Computational Biology (ECCB) to organize a joint meeting in Europe every other year. This led to the ISMB/ECCB in Vienna in 2007 that set the standard for a large-scale integrative forum for all those with interest in subjects related to computational biology.

ISCB is now focusing on expanding participation beyond North America and Europe, which has accounted for the majority of participants during the history of ISMB. One meeting in South Asia (InCoB; http://incob.binfo.org.tw/) has already been sponsored by ISCB, and another one in North Asia is going to follow. ISMB itself has also been held in Australia (2003) and Brazil (2006).

#### Multi-tracking in response to challenges posed by merging fields

ISMB/ECCB 2007 considerably expanded the breadth of the meeting. ISMB 2008 in Toronto builds on its successful predecessor by keeping what worked, and by providing more open time for spontaneous exchange and meetings. The main goal of ISMB is to contribute toward the success and enjoyment of science. In fact, scientific process is highly dependent on good communication of results and nascent ideas. Creating outlets for such communication is therefore an important catalyst for the advancement of computational biology.

The major challenge for this interdisciplinary field is that two cultures with very different ways of publishing intersect. On the one hand, computational sciences publish their most important results in rigorously reviewed proceedings of meetings; the lower the ratio between accepted/submitted papers, the more valued the publication. Publications in conference proceedings are often more highly regarded than those in peer-reviewed scientific journals. In contrast, the life sciences publish their best work in peer-reviewed journals with the highest possible impact; journals with higher impact are ranked higher, and publications are not coupled to presentations at any meeting. Open access publications attach value to the individual publication rather than to the specific vehicle. Thus, they may ultimately offer a way to bring these two publication cultures together. However, this approach does not address the needs of all members of a Society rooted firmly in both cultures.

Accepting this complex challenge already implies the need for at least four parallel tracks: two for presentations from proceedings (*Proceedings Tracks*), two for presentations from high-profile work published in refereed journals (*Highlights Tracks*). Further expansion may offer even greater opportunities to include new and relevant areas of research into the scientific program.

#### Events in light of a scientific career

**Figure 1:**
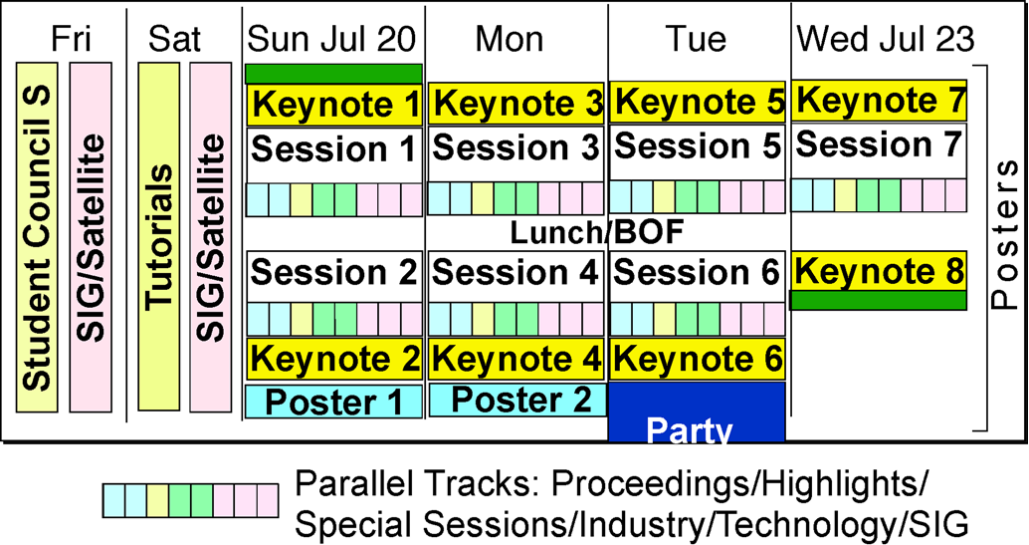
Sketch of Program for ISMB 2008 in Toronto.

One way of drawing the dividing line between the multiple events (Figure 1) is with respect to the stage of the participant in their scientific career. Young participants (students, postdocs, and young faculty) typically present *Posters* that may combine published and unpublished material and may represent very recent work. If the work has matured to the publication stage, the investigator may consider submitting to the *Proceedings Track* for novel, original research work. (We note that the acceptance rate is typically below 17%, i.e., more restrictive than most peer-reviewed journals and most computer science conferences). Most papers accepted in past ISMB proceedings were by investigators who were already established in the community. Published work with a track record of being a highlight in the field finds a home in the *Highlights Track.* One of the selection criteria for a *Highlights* talk is that the designated presenter has strong presentation skills. Thus, most presenters are already well-known in the community. An important body of work is typically the prerequisite for invitation for a *Keynote Presentation* by an accomplished researcher. Finally, the *Special Sessions* are ways to expose the field to areas and novelties that are not already integrated into the community. The extension of what was previously a track for software demonstrations to a more inclusive *Technology Track* now includes investigators from all scientific career stages.

#### Keynotes

The *Keynote Presentations* (http://www.iscb.org/ismb2008/keynotes.php) are run in a single track with no other competing session and typically attract most participants. In Toronto we will continue our pursuit of several missions for these talks: to make the marriage between computational and experimental biology more beneficial for biology, to present outstanding, exciting, and thought-provoking jewels of the biological sciences, and to celebrate the marvels uniquely represented in the world by ISCB.

Two *Keynote Presentations* feature the winners of the annual ISCB awards—the Overton Prize, in memory of Chris Overton, for outstanding investigators early in their career, and the Accomplishment by a Senior Scientist Award given to those at the zenith of their career who have made major contributions to the field of computational biology through research, education, service, or a combination of the three. This year's Overton prize goes to Aviv Regev (Broad Institute of MIT and Harvard, Cambridge, Massachusetts, United States), and the Senior Scientist award goes to David Haussler (University of California Santa Cruz and Howard Hughes Medical Institute, Santa Cruz, California, United States). The awards will be presented along with the accompanying *Keynote* addresses. In fact, the Senior Scientist will conclude the scientific presentations of ISMB on the afternoon of Wednesday, July 23. Claire M. Fraser-Liggett (Institute for Genome Sciences, University of Maryland, Baltimore, Maryland, United States will kick off the main meeting with her talk on Microbial Communities in Health and Disease. The five other *Keynotes* will be given by (in alphabetical order): David Jaffe (Broad Institute of MIT and Harvard, Cambridge, Massachusetts, United States), Hanah Margalit (Hebrew University, Jerusalem, Israel), Eugene M. Myers (Howard Hughes Medical Institute, Ashburn, Virginia, United States), Bernhard Ø. Palsson (University of California San Diego, La Jolla, California, United States), and Morag Park (McGill University, Montreal, Canada). These lectures will review recent outstanding results in areas such as Oncology, Metabolic modeling and reconstruction, transcriptional regulation, genomics, microbiology, neuronal Imaging, and new sequencing technologies.

#### Special Sessions Track

For the second year, a *Special Sessions Track* (http://www.iscb.org/ismb2008/special_sessions.php) will enable in-depth focus on emerging and important areas. Seven *Special Sessions* will cover a wide spectrum of topics, including cancer research, virology, proteomics, new generations in technologies including sequencing, mass spectrometry, flow cytometry, and cell imaging. For example, the session on *Health and Diseases in our Genomes* chaired by ISMB's honorary co-chair Thomas Hudson (Ontario Institute for Cancer Research, Toronto, Canada) will highlight the role of computational biology spanning from analyzing human genome variability to understanding disease. Other *Special Sessions* will feature the Toronto scientific community's efforts towards aspects of proteomics and disease, e.g., *Interaction Networks and Disease* chaired by Shoshana Wodak and Gary Bader (University of Toronto, Toronto, Canada) and *Structural Bioinformatics: Deciphering the Proteome* chaired by Igor Jurisica (Ontario Cancer Center, Toronto, Canada) and Ryan Lilien (University of Toronto, Toronto, Canada). Imaging techniques are introduced in *Frontiers in Cell Imaging* chaired by Robert F. Murphy (Carnegie Mellon University, Pittsburgh, Pennsylvania, United States), the challenges of tackling the interactions between organisms are summarized in *Computational Challenges and Opportunities in Host–Pathogen Systems Biology* chaired by T. M. Murali and Matthew D. Dyer (Virginia Polytechnic Institute, Blacksburg, Virginia, United States), and the very large-scale sequencing of the future is previewed in *Sequencing Thousands of Human Genomes* chaired by Francisco De La Vega (Applied Biosystems, Foster City, California, United States) and Gabor Marth (Boston College, Chestnut Hill, Massachusetts, United States).

Finally, a group of investigators involved with both ISCB and the Society's official journal, *PLoS Computational Biology*, present a perspective on the *Future of Scientific Publishing* chaired by Scott Markel (ISCB Publications Committee chair, Accelrys, San Diego, California, United States). This is one of the two events highlighting the connection between ISCB and *PLoS Computational Biology* (the other is a *Tutorial on Professional Development* given by Phil Bourne, the Editor-in-Chief of *PLoS Computational Biology*).

All *Special Sessions* will review the current state of a specialized field, and will explore growing opportunities for computational biologists and the need for new tools and methodologies.

#### Proceedings Track

Traditionally the major event at the ISMB meetings is the presentation of original papers, which are published in the *ISMB Proceedings* as a special issue of the journal *Bioinformatics*. Common practice for computer science precludes the re-publication of material published in *Conference Proceedings* in any other peer-reviewed journal or meeting. Thus, the special issue of *Bioinformatics* increases the value of these *Conference Proceedings* for the more biology-oriented members in the field. In 2008, we received 287 submissions to this track; Alfonso Valencia (CNIO, Madrid, Spain) chaired the rigorous review process modeled on the editorial review in scientific journals. He and his team of Area Chairs selected 48 (16.7%) original research papers for presentation in Toronto. This acceptance rate is slightly higher than ISMB/ECCB 2007.

One problem for the reviewers is that submissions have to be accepted or rejected given the submitted material without the conditional acceptance pending major revisions that satisfy the authors. In fact, most papers accepted by journals go through revisions and are published whenever ready; the *Proceedings*, in contrast, have to be ready by May. For the first time, the mechanism of revision for some of the submissions was used, by Alfonso Valencia and his team of experts, which accounted for the higher acceptance rate.

Papers will be presented in two parallel tracks. The paper reviewing process put increased weight on work that opens new directions and is likely to impact molecular and medical biology. All papers selected for oral presentation will be published in the conference proceedings as part of a regular online issue of *Bioinformatics* under the open-access model, i.e., will be made freely available, and, as in previous years, will be fully indexed in PubMed and by the Institute of Scientific Investigation (ISI).

#### Highlights Track

For the second year, there will be a *Highlights Track* (http://www.iscb.org/ismb2008/highlights.php) featuring recently published, high impact work. Work from highly ranked journals and from journals traditionally featuring more experimental biology is welcome. As with the other two tracks that were introduced in Vienna (*Special Sessions Track* and the *Industry Track*, below), the *Highlights Track* was very well received by many participants, as it increased diversity and added quality. This year, we received 189 proposals for this track. A diverse team of accomplished and dedicated experts accepted 61 of those for presentation in two *Highlights Tracks* in Toronto (Table 1). Papers from high-impact journals such as *Science* and *Nature*, as well as the official ISCB journal, *PLoS Computational Biology*, were submitted. In all, more than 40 journals were cited among the submissions received, ranging from *Bioinformatics* to *PNAS*, from *Cell* to *PLoS Biology*, and from *Molecular Systems Biology* to *PLoS ONE*.

**Table 1:** ISMB/ECCB History.

Year	Location	Attendees	Papers Accepted	Papers Submitted	Highlight Accepted	Highlight Submitted	Keynotes	Posters Accepted	Tutorials	SIG	Special Sessions
1999	Heidelberg, Germany	655	34	91			8	139	10	2	
2000	San Diego, US	1272	42	141			7	275	14	1	
2001	Copenhagen, Denmark	1251	38	180			6	345	14	6	
2002	Edmonton, Canada	1624	42	207			8	494	15	6	
2003	Brisbane, Australia	927	48	342			8	414	16	5	
*2004*	Glasgow, UK *	*2136*	*67*	*496*			*8*	*952*	*14*	*8*	
2005	Detroit, US	1731	56	426			8	622	14	8	
2006	Fortaleza, Brazil	880	67	407			8	556	12	4	
*2007*	Vienna, Austria *	*1752*	*63*	*418*	*63*	*215*	*10*	*1096*	*14*	*8*	*7*
2008	Toronto, Canada		48	287	61	189	8		10		8

*: Meetings organized jointly by ISCB and ECCB, with their data in italics.

#### Industry Track

The *Industry Track* (http://www.iscb.org/ismb2008/industrytrack.php) will run for the second year and is for talks that are more relevant and interesting for participants from industry. Chaired by Reinhard Schneider (EMBL, Heidelberg, Germany), this track introduces a new forum for the meeting of academia and industry in a venue that highlights innovative applications and practical impact studies of Life Science Informatics. Each selected talk will describe a scientific problem from a business perspective, including the approach used to address the problem, the current state of the project, an evaluation of the benefits, and plans for future developments. These presentations are tailored to give attendees the opportunity to view scientific approaches through an industrial lens, which may prove especially valuable to young researchers' understanding of the use of Life Science Informatics in the business sector.

#### Technology Track (previously Demo Track)

Demonstrations of software have also become an integral part of the ISMB and ECCB conference series, and are now also offered at other bioinformatics conferences. Demos allow academic institutions as well as for-profit organizations to showcase their software and/or hardware in a hands-on format to audiences of up to 50 participants. The *Demo* sessions have proven to be in high demand, and have consistently added a valuable aspect of the conference for both presenters and attendees alike. These traditional demonstrations are currently being selected by a team of experts chaired by Rodrigo Lopez (EBI, Hinxton, England) and a team of experienced colleagues.

For the Toronto meeting, we decided to extend the concept of demonstrations by integrating other events that had no traditional place in the ISMB framework. The change in the name from *Demo* to *Technology Track* reflects the following extensions and novelties. Firstly, we will give institutions and research consortia the opportunity to reserve a block of slots and use this to evolve the concept of a demonstration into a series of synchronized demonstrations. The second is another Toronto pilot: in addition to the traditional tutorials, we will introduce interactive, hands-on workshops for freely available software tools and methods. The ones chosen for the pilot reflect the wide range of popular packages and the range of activity in Computational and Systems Biology. These workshops will be more comprehensive than the current “demos” and give attendees an opportunity to bring their own data, laptops, and problems for practical learning.

#### Posters: Continuous Display

For the second time, all *Posters* (http://www.iscb.org/ismb2008/posters.php) accepted for presentation will be on exhibit for the entire meeting. Over the years, ISMB has grown to a size that has made it impossible to display all accepted posters during the entire conference. Yet, posters bring enormous value to the conference. The Metro Toronto Convention Center hosting ISMB 2008 has the capacity to accommodate more than 1,000 *Posters* on display at the same time. Therefore, we are especially pleased to ease the viewing of *Posters* by allowing all attendees the opportunity to view and absorb this abundance of research in a single venue. Two *Poster Author Sessions*, carved out of the evening program, will provide an opportunity to further explore posters of specific interest to any attendee: one evening for even- and one for odd-numbered *Posters* will be available. Marco Punta (Columbia University, New York, US) chairs the selection of *Posters* in Toronto.

#### Tutorials, SIGs, and Students' Symposium

An increasingly important aspect of ISMB is the series of smaller meetings that accompany the main event, namely the Satellite and Special Interest Group Meetings (*SIGs;* http://www.iscb.org/ismb2008/sigs.php). For Toronto, the event is coordinated by a group of experts chaired by Hershel Safer (Weizmann Institute, Rehovot, Israel). Five meetings will run for two days (July 18–19) and four for one day. The two-day meetings are: *3Dsig: Structural Bioinformatics & Computational Biophysics*, the *Bioinformatics Open Source Conference (BOSC)*, *BioPathways*, *Alternative Splicing*, and the *Joint AFP-Biosapiens SIG*. Two of the one-day meetings will precede the main meeting: *BioLink* (July 18) and *Next Generation Sequencing* (July 19). Two others will occur during the main meeting: *Bio-Ontologies* (July 20), and *Genome-Scale Pattern Analysis in the Post-ENCODE era* (July 21).

From the beginning, an integral part of ISMB has been the *Tutorials* (http://www.iscb.org/ismb2008/tutorials.php) that ran the day before the main meeting. Janet Kelso (MPI for Evolutionary Anthropology, Leipzig, Germany) chairs this event. In the attempt to shorten the overall meeting, the *Tutorials* will be given in parallel with some of the SIGs on July 19.

For the first time, the *ISCB Student Council Symposium* (SCS4; http://www.iscb.org/ismb2008/scs4.php) on July 18 chaired by Lucia Peixoto (University of Pennsylvania, Philadelphia, US) and Amr Abuzeid (Queen's University, Toronto, Canada), will run in parallel with the first day of two-day SIGs preceding ISMB. Each of these pre-conference meetings offers additional opportunities to learn and network with a specific group of peers of similar interests and goals.

#### ISMB Expo

Another pillar of ISMB is the *Exposition* that runs through the entire main meeting (July 20–23). The *ISMB Expo* has consistently proven to be highly successful for the exhibiting companies featuring products and services of specific value to the computational biology community. Similarly, ISMB attendees take full advantage of the opportunity to meet one-on-one with the conference sponsors and exhibitors to catch the latest updates and information on valuable software, hardware, publications, and tools of the trade. Overall, the *Expo* is another avenue focused on the importance of industry in science, and many of the exhibitors present talks in the *Industry Track* to detail the problems, applications, benefits, and future plans being addressed within their companies.

#### More than just science

Attendees traveling with family members and/or children will find this year's event offers a variety of optional tours and activities during the meeting, and extended tours on dates surrounding the conference. Specifically for those traveling with children, we are working on providing childcare options and information that would enable parents to attend the meeting while children are safely entertained.

Two other Toronto novelties try to expand into different realms: The first is *Visual Reflections on Science* (http://www.iscb.org/ismb2008/vrs.php) organized by the ISCB Student Council (ISCBSC; http://www.iscbsc.org/) and chaired by Milana Frenkel-Morgenstern (Weizmann Institute, Rehovot, Israel). A space has been set aside for the presentation of ideas that reflect upon science from a very different perspective—perhaps more art than science. Finally, we will celebrate the 16^th^ ISMB with an Off-Site Dinner followed by the high energy Matrix Party; this event will happen in Toronto's *Liberty Grand* (http://www.libertygrand.com/) entertainment complex.

#### Challenges of numbers

**Figure 2:**
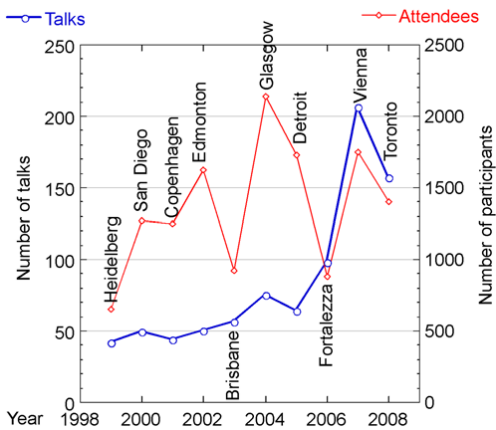
Increasing Breadth of ISMB.

Many of us may remember our first ISMB and our surprise at how many colleagues we wished to connect with attended the meeting. ISMB connects an interdisciplinary scientific community through a single, international point of contact. ISMB has been growing in terms of its breadth of coverage and the number of presentations (Figure 2).

At our current size, it becomes extremely challenging to identify venues that are big enough to host meetings and at the same time small enough to foster informal communication and a more intimate feel. While many countries have venues that would allow meetings of between 1,000 and 5,000 participants, such venues often do not permit the level of full-scale parallelism that ISMB has grown into (e.g., since we cannot tell upfront which track will be most visited, we currently need to reserve seven rooms each of which can seat more than 700 participants). The Metro Convention Center in Toronto is one of the few sites that appear to be ideal for the goals of ISMB.

ISMB 2008 is expected to draw more than 1,400 attendees to Toronto to take part in the world's largest and most scientifically comprehensive bioinformatics meeting of the year. ISCB members gain significant *ISMB 2008 Registration* (http://www.iscb.org/ismb2008/registration.php) discounts, while non-members are offered a complimentary one-year-membership as part of their higher non-member fees. The early bird registration discount period ended June 4 for all registration categories, although registration will remain open through the conference dates.

We hope to welcome all of you in Toronto.

